# Fourth Day

**Published:** 1881-06

**Authors:** 


### FOURTH DAY—FRIDAY, MAY 6, 1881.

The Association was called to order by the President.

Prayer by Rev. C. H. Read, of the Grace Street Presbyterian Church.

The following committee was announced on Clinical Observations and Records: Dr. N. S. Davis, Chicago; Dr. J. M. Toner, Washington, D. C.; Dr. H. O. Marcy, Boston, Mass.; Dr. W. H. Geddings, Aiken, S. C.; Dr. S. M. Bemis, New Orleans.

The Association then resumed the consideration of the amendment of the Code of Ethics.

Dr. Billings then offered the following substitute for the original:

"It is not in accord with the interest of the public or the honor of the profession that any physician or medical teacher should examine or sign diplomas or certificates of proficiency for, or otherwise be specially concerned with, the graduation of persons whom they have good reason to believe intend to support and practice any exclusive and irregular system of medicine."

The substitute was adopted.

The Committee on Nominations reported the following additional officers, who were elected:

*Section on Practice of Medicine.*—Chairman, Dr. J. A. Octerlony, Kentucky; Secretary, Dr. Deering J. Roberts, Tenn.

*Section on Surgery and Anatomy.*—Chairman, Dr. J. C. Hughes, Iowa; Secretary, Dr. William A. Byrd, Illinois.

*Section on Obstetrics.*—Chairman, Dr. H. O. Marcy, Massachusetts; Secretary, Dr. C. V. Mottram, Kansas.

*Section on Medical Jurisprudence and State Medicine.*—Chairman, Dr. A. L. Gihon, Washington, D. C.; Secretary, Dr. J. H. Sears, Texas.

*Ophthalmology, Otology and Laryngology.*—Chairman, Dr. D. B. St. John Roosa, New York; Secretary, Dr. J. Solis Cohen, Philadelphia, Pa.

*Diseases of Children.*—Chairman, Dr. S. C. Busey, Washington, D. C.; Secretary, Dr. William Lee, Baltimore, Md.

*Dentistry.*—Chairman, Dr. D. H. Goodwillie, New York; Secretary, Dr. P. B. Brophy, Illinois.

*Judicial Council.*—Dr. S. N. Benham, Pennsylvania; J. M. Toner, Washington, D. C.; D. A. Linthicum, Arkansas; William Brodie, Michigan; H. D. Holton, Vermont; A. B. Sloan, Missouri; R. Beverly Cole, California.

Dr. D. S. Reynolds, of Kentucky, delivered the address of the chairman of the Section on Ophthalmology, etc., which was appropriately referred. The reports of the Committees on Necrology and Secretaries of Sections were now submitted.

Dr. William M. Beech, of Ohio, chairman, submitted the report of the committee appointed on official position of members of the medical staff of the army and navy, in which he asked that the committee be continued. In doing so, he argued that the principle of promotion which prevails among army officers should prevail the same as it does in the other departments of the Government.

He spoke earnestly upon the subject, and was frequently applauded. He complained that while officers of the army and navy were retired on half pay, surgeons in the army and navy, who had been in the service for years, did not have the same recognition from the Government. He showed that this principle extended to the judiciary as well.

Dr. Brown, United States Navy, suggested that the report was wrong in its title, complaining of the lack of official recognition of medical officers of the army and navy. He thought the matter of official recognition would right itself.

Dr. Billings, United States Army, concurred in what Dr. Brown said, and thought the subject had better drop.

Dr. Beech was willing to strike out the language relating to official recognition, but he thought some legislation should be enacted by Congress to secure equal preferment with other officers of the army and navy.

On motion of Dr. Davis the matter was laid on the table.

The Convention adopted the following:

*Resolved*, That the thanks of this Association are hereby tendered to the Committee of Arrangements of this Association for the faithful attention they have given to their duties and requirements; to the medical profession and citizens of Richmond for their hospitality and endeavors to make the time spent by us while here pleasant and agreeable; to Drs. McCaw and McGuire for the elegant special entertainment given by them at the Westmoreland Club; to Mr. McClure, Superintendent of the Telephone Company, for special facilities given the Committee of Arrangements and the Association; to Vice-President Parsons, of the Richmond and Alleghany Railroad, for his kind invitation for a ride on his road, in order to show us the interior of the State of Virginia; to Mr. Powell, manager of the Richmond Theater; to the managers of the Mozart Association, and all others who have contributed to our pleasure and comfort; to the press, and especially their reporters, in giving such a full *résumé* of the proceedings in the daily papers; to the railroad companies generally who have so liberally reduced the rates of transportation for our benefit and any other modes of conveyance that have so contributed; to Mr. Valentine for his kind invitation to his studio.

*Be it especially resolved*, That our thanks are particularly due to the ladies of Richmond, for their attention and kind interest in making our sojourn so very pleasant and agreeable.

The hour having arrived for adjournment, Dr. N. S. Davis, of Chicago, alluded to the very harmonious session which had been held, and complimented the retiring President for his efficiency in the discharge of his duties.

The retiring President (Dr. Hodgen) returned his thanks to his friends in Richmond who had so kindly entertained the members of the Association. He was reminded that his ancestry first breathed the air of heaven in this delightful country, and as he remembered the bright smile of his now dear, dear mother, he would in future remember the impression that had been made upon him by the people of Richmond, Va. [Applause.]

The Association was then declared adjourned *sine die*.

